# Anti-Melanoma Capability of Contactless Cold Atmospheric Plasma Treatment

**DOI:** 10.3390/ijms222111728

**Published:** 2021-10-29

**Authors:** Dayun Yan, Qihui Wang, Xiaoliang Yao, Alisa Malyavko, Michael Keidar

**Affiliations:** 1 Department of Mechanical and Aerospace Engineering, George Washington University, Washington, DC 20052, USA; qwang52@gwmail.gwu.edu (Q.W.); xiaoliang_yao@gwmail.gwu.edu (X.Y.); 2 School of Medicine and Health Science, George Washington University, Washington, DC 20052, USA; alisamalyavko@gwmail.gwu.edu

**Keywords:** melanoma treatment, cold plasma, non-invasive, contactless treatment

## Abstract

In this study, we demonstrated that the widely used cold atmospheric plasma (CAP) jet could significantly inhibit the growth of melanoma cells using a contactless treatment method, The flow rate of helium gas was a key operational parameter to modulate electromagnetic (EM) effect on melanoma cells. Metal sheets with different sizes could be used as a strategy to control the strength of EM effect. More attractive, the EM effect from CAP could penetrate glass/polystyrene barriers as thick as 7 mm. All these discoveries presented the profound non-invasive nature of a physically based CAP treatment, which provided a solid foundation for CAP-based cutaneous/subcutaneous tumor therapy.

## 1. Introduction

Cancer is a significant threat to human health [1]. The search for topical, local, and non-invasive treatments for managing cancers is a mainstay in modern medicine, especially for superficial tumors such as melanoma or squamous cell carcinoma [2]. Obviously, due to the cutaneous or mucosal nature of melanoma, it may be one of the most promising candidates for CAP-based cancer therapy [3]. Even for melanoma, however, the tumorous tissue will not directly expose to plasma without the help of surgery, not to mention other deep tumors. Thus, there is an urgent need to explore the non-invasive potential of CAP treatment, particularly contactless treatment based on physical factors. 

As a non-equilibrium plasma, CAP acts through both chemical and physical factors [4]. Reactive species, such as reactive oxygen species (ROS) and reactive nitrogen species (RNS) have been regarded as the main players in the biological effect of CAP in many branches, including cancer treatment, wound healing, microorganism sterilization, agriculture, as well as environmental engineering [5,6,7,8,9,10,11,12]. To date, the physical effects of CAP have been ignored, probably mainly because of the absence of direct experimental evidence [13,14,15]. 

Typically, CAP will generate at least three physical effects: thermal, ultraviolet (UV), and EM. Recently, we have demonstrated that EM effect rather than thermal and UV effects could trigger strong cell death in several cancer cell lines, including glioblastoma cells, lung cancer cells, as well as bladder cancer cells [16,17,18]. Such a physically triggered cell death is obvious necrosis, characterized by a fast cytosol aggregation and noticeable bubbling on the cellular membrane [16,17,18]. Bubbling is due to the leakage of cellular solutions into the extracellular environment.

An attractive feature of physically based treatment is that the EM effect in CAP could penetrate a physical barrier such as the bottom of multi-well plates or dishes with a thickness around 1 mm to kill cancer cells [16,17,18]. These physical barriers definitely will block all chemical factors such as long-/short-lived reactive species. The penetration depth of EM effect is still unknown. Here, we demonstrated that a strong anti-melanoma effect could be achieved by a contactless approach. A strong anti-melanoma effect could still be observed even when there was a 7 mm thick glass and polystyrene material barrier between plasma and cells. The results of this study could be used as guidelines to design novel CAP sources based on their physical effect. 

## 2. Results

### 2.1. Guideline to Observe Necrosis

**Figure 1:**
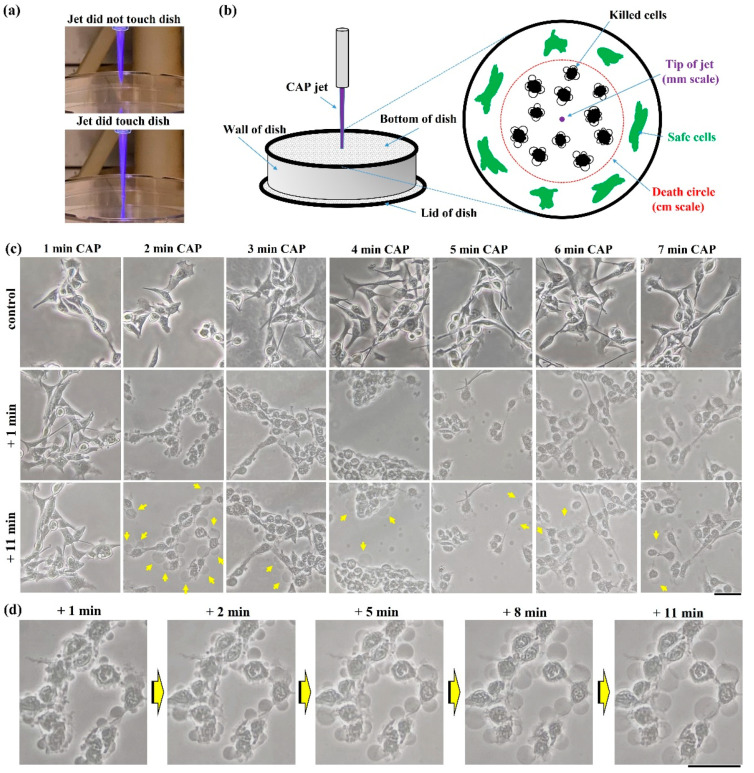
Cell death in physically based CAP treatment: (**a**) Photos of the CAP jet’s tip; (**b**) schematic illustration of microscopic observation on physically triggered cell death. The damaged area by physical effect is much larger than the size of the CAP jet’s tip. All microscopic observations focused on the center of ‘death circle’; (**c**) morphological changes in the B16F10 cells. (**d**) Zoomed-in images of cells after 2 min of treatment. Yellow arrows marked the bubbles on cellular membranes. The detached bubbles have not been marked. Experimental details were briefly illustrated here: B16F10 cells were seeded with a density of 1 × 10^5^ cells/mL on a 35 mm glass-bottom dish for 24 h before treatment. Helium flow rate was 1.53 lpm. The gap between the nozzle and the bottom surface of the 100 mm dish was 27 mm. Fresh medium (1.5 mL) was added in the dish immediately after treatment. Photos were taken at 1 or 11 min after treatment. Scale bar = 50 μm. All photos were taken by a Nikon TS100 inverted phase-contrast microscope.

As mentioned above, bubbling was the most obvious feature to discriminate the physically triggered necrosis from others. First, we demonstrated an ideal condition to perform microscopic observations on bubbling. Take the cell culture dish as an example, treatment was performed on the bottom of an inverted (upside-down) dish (35 mm or 100 mm) (Figure 1a). In this case, the CAP jet’s tip had a diameter of less than 1 mm when it did not contact the dish bottom. Even when the CAP jet’s tip touched the dish, the contact area diameter was still at a millimeter-scale range. In contrast, such a small-scale touch between plasma and dish could affect a much bigger area at a centimeter-scale range (Figure 1b). All physically damaged cancer cells were in a circular area, named the ‘death circle’. In this study, we only focused on the cells’ morphological changes in the middle of the ‘death circle’.

The B16F10 cells experienced drastic necrosis after treatment. There was no noticeable cellular change after 1 min of treatment. However, when treatment was extended to 2 min or longer, cells experienced a similar change, mainly including cytosol aggregation and bubbling on the cellular membrane (Figure 1c,d). The bubbling was due to the leakage of cellular solutions into the extracellular environment. The most apparent bubbling phenomena could be observed when treatment time was 2 min. When treatment time increased, the observable bubbling decreased and more bubbles appeared in the extracellular space, due to the detachment or direct damage on the formed bubbles. To observe the strongest bubbling phenomena, a 2 min CAP treatment was recommended in this study. 

### 2.2. Flow Rate’s Effect

**Figure 2:**
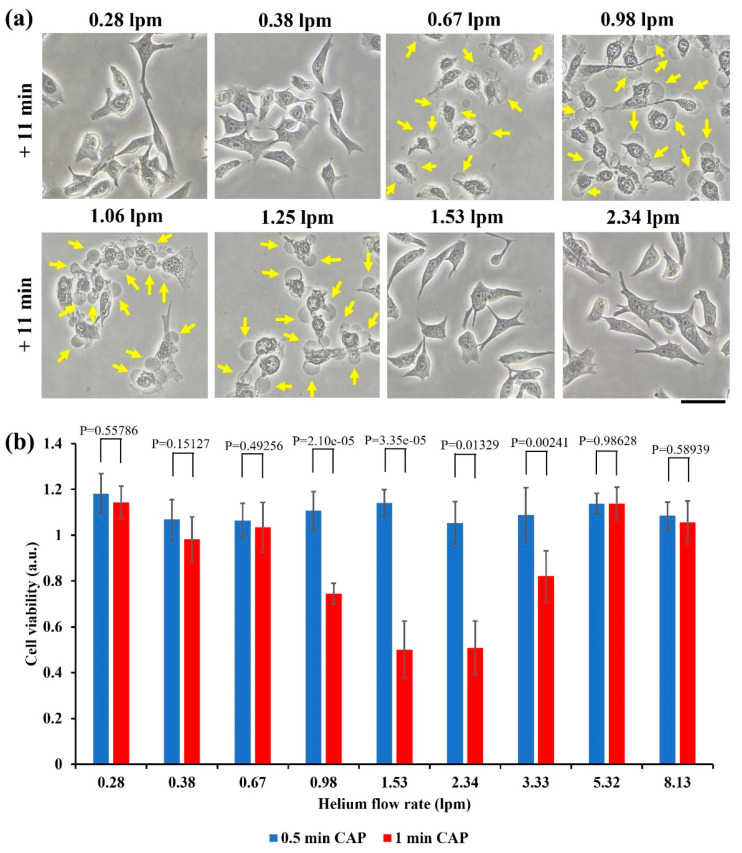
Helium flow rate drastically affects physically triggered necrosis: (**a**) Morphological changes of the B16F10 cells in the 100 mm dish after 3 min of CAP treatment with different flow rates. Yellow arrows marked some bubbles on the cellular membranes. “+ 11 min” means the bright field imaging was taken at 11 min after treatment. Scale bar = 50 μm. Experimental details were briefly illustrated here: B16F10 cells were seeded (7.5 × 10^4^ cells/mL) on a 100 mm dish (10 mL) for 24 h before treatment. Fresh medium (10 mL) was added in the dish immediately after treatment. The gap between the nozzle and the bottom surface of dish was 27 mm. All photos were taken using a Nikon TS100 inverted phase-contrast microscope. (**b**) Cell viability assay of the B16F10 cells in the 12-well plate. Data were presented as the mean of at least three independent experiments. Statistical significance was calculated via one-way analysis of variance (ANOVA) followed by Bonferroni post hoc test for multiple comparisons. Experimental details were briefly illustrated here: Following the protocols in Methods and Materials, the B16F10 cells (1 mL/well) were seeded (7.5 × 10^4^ cells/mL) in a 12-well plate and cultured 24 h before treatment. The gap between the nozzle and the bottom surface of plate was 25 mm. After treatment, 1 mL/well of fresh medium was immediately supplied in a 12-well plate, and cells were cultured three days before MTT assay.

The flow rate of noble gas such as helium is an important operational parameter to modulate both chemical and physical properties in CAP. A higher flow rate tends to generate more reactive species, particularly, long-lived reactive species such as H_2_O_2_ [19,20,21]. In contrast, the impact of helium flow rate on the CAP’s EM effect shows a completely different trend. When helium flow rate increased from 0.28 lpm to 2.34 lpm, the cellular response (necrosis) did not show a simple proportional response. As shown in Figure 2a, the noticeable necrotic features occurred only when flow rate was at a middle range, from 0.67 lpm to 1.25 lpm. Neither too small nor too large flow rate would cause these necrotic cellular changes.

In addition, the cell viability assay provided a similar trend quantitatively. As shown in Figure 2b, a similar trend was observed in the MTT assay. The strong growth inhibition effect would be observed only in the middle range of helium flow rate, from 0.98 lpm to 3.33 lpm. Different from the treatment performed on the 100 mm dish, the maximum growth inhibition occurred when the flow rate was 1.53 lpm or 2.34 lpm. Thus, specific experimental conditions such as the size of dish might affect the specific physical effect, although the general trend did not change. 

### 2.3. ROS Scavenger Cannot Inhibit Necrosis

Rise of intracellular ROS is key cellular response to the chemically based CAP treatment, or more precisely, to the CAP-originated reactive species [22]. Pretreatment of intracellular oxidant scavenger such as N-acetylcysteine (NAC) can effectively inhibit the reactive species-induced cellular response, including an increase in ROS, DNA damage, mitochondrial damage, and cell death mainly apoptosis [23]. Here, the B16F10 cells were also pretreated by NAC-containing RPMI (1, 5, and 10 mM) for 60 min before the physically based CAP treatment on the bottom of an inverted dish. 

**Figure 3:**
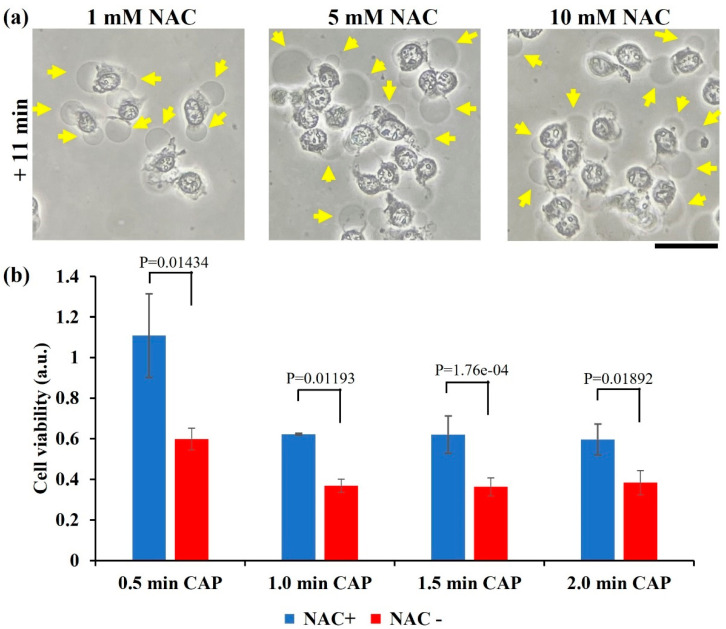
NAC pretreatment did not eliminate physically triggered necrosis. (**a**) Morphological changes in the NAC pretreated B16F10 cells after 3 min of CAP treatment. Yellow arrows marked some bubbles on the cellular membranes. “+ 11 min” means the bright field imaging was taken at 11 min after treatment. Experimental details were briefly illustrated here: B16F10 cells were seeded (7.5 × 10^4^ cells/mL) on a 100 mm dish (10 mL) for 24 h before treatment. Helium flow rate was 1.25 lpm. The gap between the nozzle and the bottom surface of dish was 27 mm. Fresh medium (10 mL) was added in the dish immediately after treatment. Scale bar = 50 μm. All photos were taken using a Nikon TS100 inverted phase-contrast microscope. (**b**) Cell viability assay. “NAC+” and “NAC−” represent with and without NAC pretreatment (20 mM), respectively. Data were presented as the mean of three independent experiments. Statistical significance was calculated via one-way analysis of variance (ANOVA) followed by Bonferroni post hoc test for multiple comparisons. Experimental details were briefly illustrated here: Following the protocols in Methods and Materials, cells (1 mL/well) were seeded (0.5 × 10^4^ cells/mL) in a 12-well plate and cultured 24 h before treatment. Helium flow rate was 1.53 lpm. The gap between the nozzle and the bottom surface of plate was 25 mm. After physically based treatment, 1 mL/well of fresh medium was immediately supplied in a 12-well plate, and cells were cultured three days before the MTT assay.

Pretreatment of NAC did not effectively inhibit the anti-melanoma capability of CAP treatment. Typical necrotic features such as cytosol aggregation and strong bubbling still appeared on the B16F10 cells (Figure 3a). Furthermore, a cell viability assay was performed on the NAC pretreated B16F10 cells in a 12-well plate. As shown in Figure 3b, physically based treatment caused a strong killing effect on the B16F10 cells, with or without NAC pretreatment. However, NAC pretreatment did weaken growth inhibition in different levels as compared with the cells without NAC treatment. Particularly, when treatment time was just 0.5 min, NAC pretreatment inhibited growth inhibition by nearly 50%. Thus, physical factor might still affect the intracellular redox balance with a weak level. These results again demonstrated that physically based CAP treatment exerted a totally different mechanism to cause necrosis as compared with previous studies based on the reactive species triggered apoptosis. 

### 2.4. Blockage Effect of Copper Sheets

The CAP jet’s tip temperature was slightly less than 40 °C. A heat reflection film (Design Engineering, 010462) was used to cover a 5 × 5 well area centered at the well “6D” on a 96-well plate to block possible thermal effect from bulk CAP. Experimental details were briefly illustrated here: B16F10 cells (6 × 10^4^ cells/mL) were seeded in a 96-well plate (100 μL/well) and cultured for 24 h before treatment. The gap between the nozzle and the bottom surface of the 96-well plate was 25 mm. Helium flow rate was 1.53 lpm. Following the protocols in Methods and Materials, the MTT assay was performed 24 h after treatment. 

**Figure 4:**
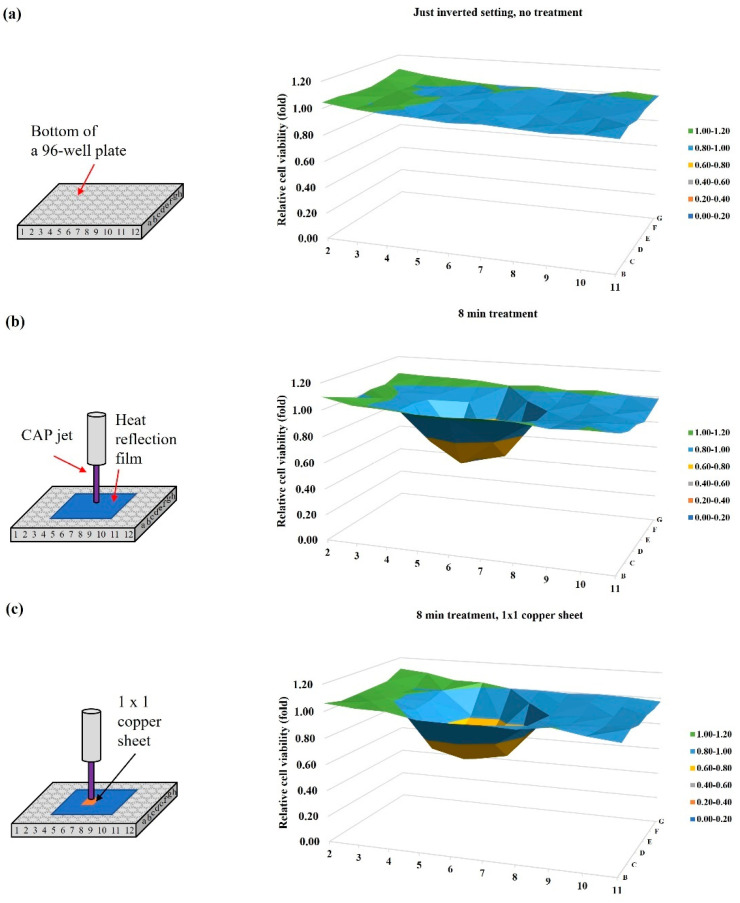

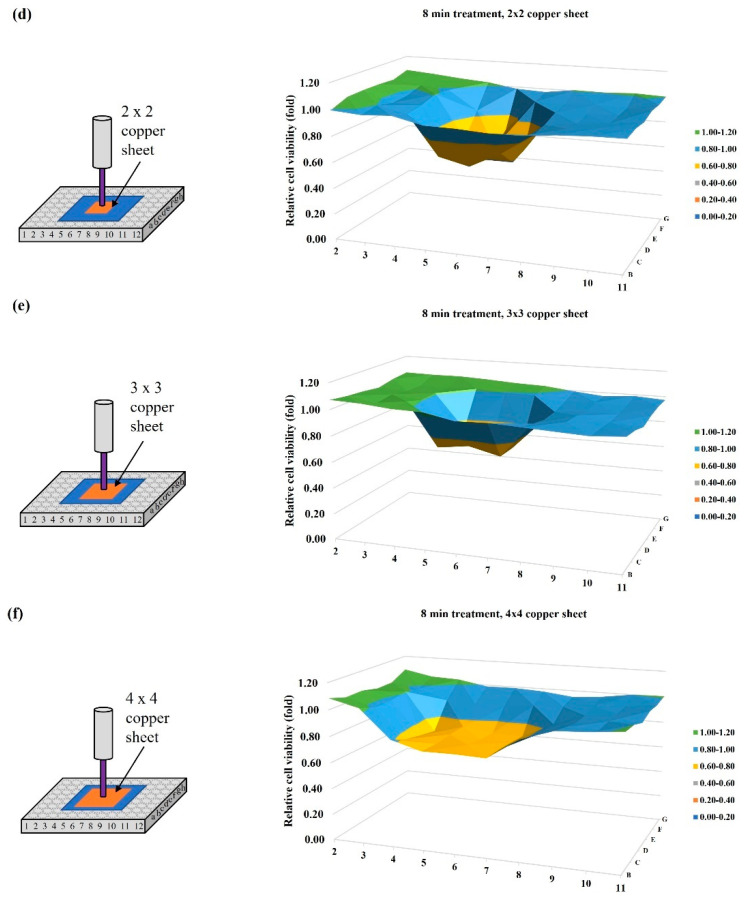
Size of copper sheet affected the physically based anti-melanoma effect. Schematic illustration and 3D cell viability map are present in the left and right panel, respectively. (**a**) Control, an inverted setting for 8 min, without any treatment; (**b**) a 5 × 5 well heat reflection film covered the whole middle 25 wells; (**c**) a 1 × 1 well copper sheet above a 5 × 5 well heat reflection film. (**d**) A 2 × 2 well copper sheet above a 5 × 5 well heat reflection film; (**e**) a 3 × 3 well size copper sheet above a 5 × 5 well heat reflection film; (**f**) A 4 × 4 well size copper sheet above a 5 × 5 well heat reflection film. For experimental groups (**b**–**f**), the CAP treatment lasted 8 min. CAP treatment has been independently repeated for four times.

Just 8 min of the inverted setting of the 96-well plate without the coverage of bulk medium on cells would not cause growth inhibition on the B16F10 cells (Figure 4a). The heat-reflection sheet did not inhibit physically based killing effect on melanoma cells (Figure 4b). Here, we only focused on the role of EM effect on the physically triggered cell death. To block EM effect, an ideal conductive material such as metal is desirable. Four different sizes of square copper sheet (1 × 1, 2 × 2, 3 × 3, 4 × 4 well sizes) were purchased from McMasterCARR (9709k704). The thickness of copper sheet was 1 mm. N × N means a square copper sheet could cover N2 wells. For example, a 3 × 3 copper sheet could cover nine wells. The copper sheet was just above the heat-reflection film during a single treatment (Figure 4c–f). The copper sheet was centered at the well “6D”, as was the CAP jet. The 1 × 1 well copper sheet almost does not weaken the physically based killing effect. A weak inhibition was observed in the case using a 2 × 2 well copper sheet. When the size of copper sheet increased, stronger inhibition could be observed. When the size of copper sheet was adequately large, the physically based growth inhibition could be completely inhibited [17].

### 2.5. Penetration Capability of Physically Based Treatment 

**Figure 5:**
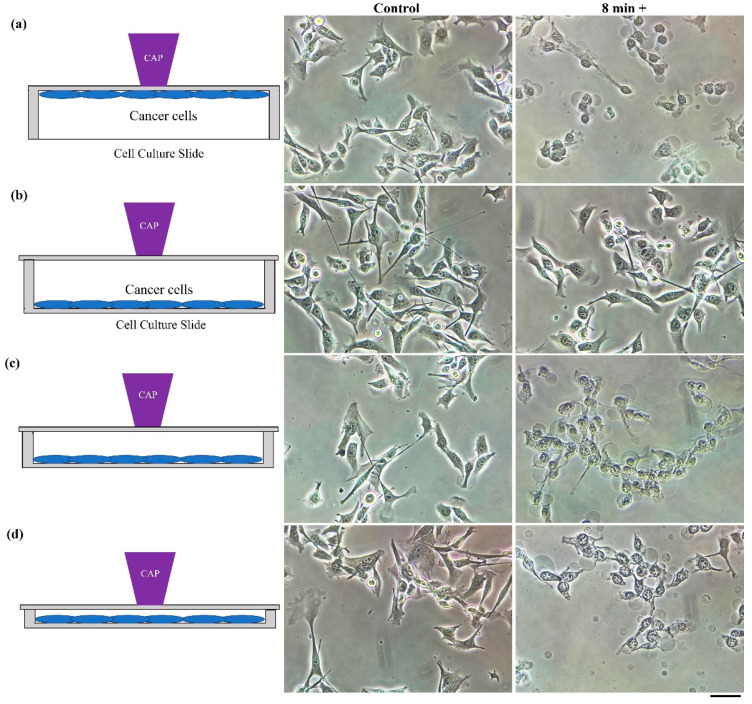
The gap between plasma and cells is a crucial factor for anti-melanoma effect. Schematic illustration and bright-field images of cells are shown in the left and right panels, respectively. “8+” meant photos were taken 8 min after treatment of 2 min. (**a**) Treatment on the bottom of an “upside-down” cell culture chamber slide (gap = 1 mm); (**b**) treatment on the lid of a cell culture chamber slide (gap = 13 mm); (**c**) treatment on the lid of a cell culture chamber slide (gap = 5 mm); (**d**) treatment on the lid of a cell culture chamber slide (gap = 2 mm). Experimental details were briefly illustrated here: The B16F10 cells were seeded (3 mL, 5 × 10^4^ cells/mL) in a cell culture chamber slide for one day before treatment. Helium flow rate was 1.53 lpm. Immediately after treatment, a fresh medium (3 mL) was supplied to the culture chamber slide, followed by bright-field imaging. All photos were taken using a Nikon TS100 inverted phase-contrast microscope. Scale bar = 50 μm.

A physically based killing effect could be observed when treatment was performed in another direction as well. The natural sensitivity of EM effect to the distance determines the sensitivity of physical effect to the gap between the CAP source and targets. A strong killing effect could be observed when treatment was performed on the bottom of an inverted 12-well plate. In contrast, a treatment just above the lid of the 12-well plate would not cause any impact on the cell viability of the B16F10 cells. Such a drastic difference should be due to the large gaps between the lid and the cells. We further investigated the gap effect via using cell culture chamber slides (NST230111), in which the gap could be changed. Similarly, a strong physically triggered necrosis could be observed when treatment was performed on the bottom of the “upside-down” slide (Figure 5a). When treatment was performed on the slide’s lid, the physically triggered necrosis did not occur (Figure 5b). When the gap between the lid and the cells gradually decreased, a strong necrosis could be finally observed (Figure 5c,d). Thus, to observe the physicallybased killing effect, it is not necessary just from the bottom of a dish or a plate. 

Effective delivery of reactive species across biological samples such as the skin is pivotal in plasma medicine [24]. Nonetheless, whether reactive species, particularly, short-lived reactive species, can deeply cross the skin or other epithelial tissue is still an open question. As compared with chemically based CAP treatment, physically based CAP treatment has an obvious advantage, i.e., the cross-barrier capability. Such a property may be due to the effective penetration of EM effect across some materials, particularly, dielectric materials. In addition, dielectric materials such as glass have been widely used in CAP sources, particularly in CAP jet sources. The thickness of glass may affect the physical effect of CAP on biological samples. 

**Figure 6:**
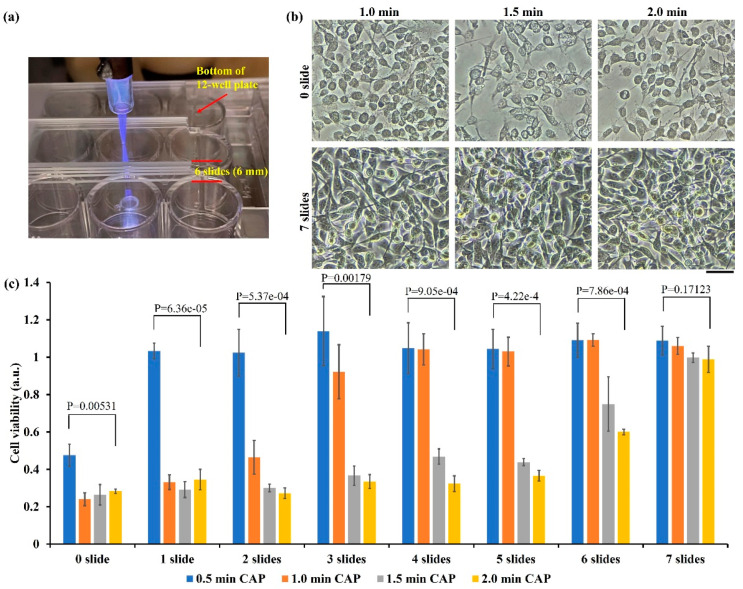
Penetration of EM effect through glass barriers: (**a**) A representative photo of treatment on glass slides above the bottom of a 12-well plate. A single slide had a thickness of 1 mm; (**b**) representative bright-field images (1 day after treatment) of the CAP-treated B16F10 cells. “0 slide” means treatment was performed directly on the bottom of the 12-well plate. “7 slides” means treatment was performed on seven glass slides above the bottom of the 12-well plate. Scale bar = 50 μm. Experimental details were briefly illustrated here: Following the protocols in Methods and Materials, the B16F10 cells (1 mL/well) were seeded (0.5 × 10^4^ cells/mL) in a 12-well plate and cultured 24 h before treatment. Helium flow rate was 1.53 lpm. After treatment, 1 mL/well of fresh medium was immediately supplied in the 12-well plate, and cells were cultured three days before the MTT assay. (**c**) Physically based treatment could penetrate six glass slides to affect the B16F10 cells. Data were presented as the mean of three independent experiments. Statistical significance was calculated via one-way analysis of variance (ANOVA) followed by Bonferroni post hoc test for multiple comparisons.

Here, we investigated the penetration capability of physically based treatment. As shown in Figure 6a, the physically based treatment was performed on a standard glass slide (1 mm thickness) just above the bottom of an “upside-down” 12-well plate. We tested the maximum glass thickness to completely block the physically based killing effect. Some representative bright-field images are shown in Figure 6b. Typical physically triggered necrotic features could be easily observed in the treatment performed directly on the bottom of the 12-well plate (0 slide). Such necrotic features would not be observed when seven slides covered the bottom of 12-well plate. The MTT assay provided quantitative information. As shown in Figure 6c, the physically based treatment caused a strong killing effect on the B16F10 cells in the presence of one–six glass slides. Even in the thickest case with six slides (6 mm thick), 40% growth inhibition could be achieved when treatment lasted 2 min. Regarding the bottom thickness of plate (1 mm thick), the EM effect from CAP could penetrate 7 mm of dielectric materials and strongly kill B16F10 cells. 

**Figure 7:**
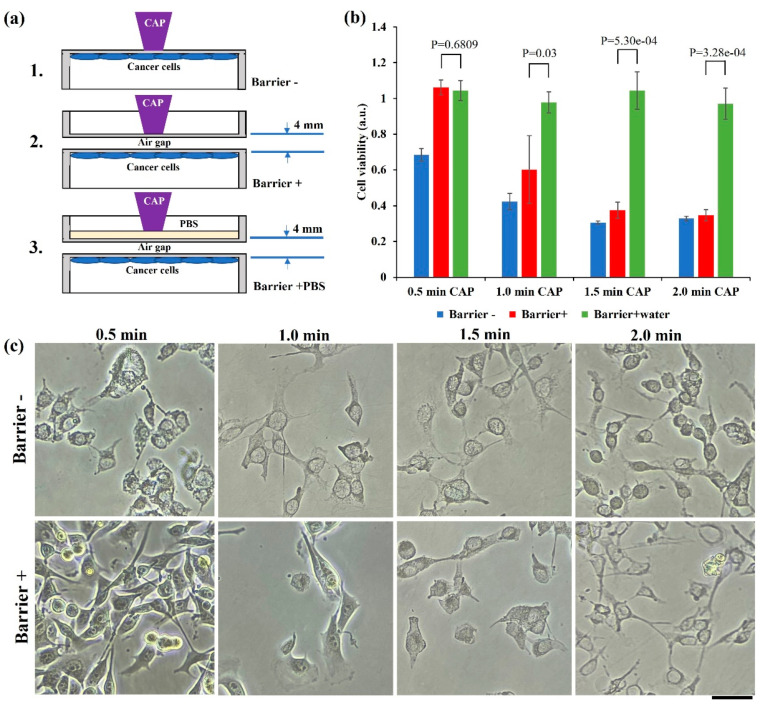
Water layer blocked physically based anti-melanoma effect: (**a**) Schematic illustration of the experimental design; (**b**) water completely inhibited the anti-melanoma effect. Experimental details were briefly illustrated here: Following the protocols in Methods and Materials, the B16F10 cells (1 mL/well) were seeded (0.5 × 10^4^ cells/mL) in a 12-well plate and cultured 24 h before treatment. Helium flow rate was 1.53 lpm. After treatment, 1 mL/well of fresh medium was immediately supplied in the 12-well plate and cells were cultured 3 days before MTT assay. Data were presented as the mean of three independent experiments. Statistical significance was calculated via one-way analysis of variance (ANOVA) followed by Bonferroni post hoc test for multiple comparisons. (**c**) Representative microscopic imaging (bright field) of cells 1 day after treatment. Scale bar = 50 μm.

Water is a crucial factor to affect the biological effect of physically based CAP treatment, which may be due to the blockage of EM effect particularly at a mm wavelength range by water. For chemically based CAP treatment, water layer facilitates transformation and diffusion of short-lived reactive species in gas phase into long-lived reactive species in liquid phase [25]. We first demonstrated that a water layer could completely block the physically based killing effect using an “upside-down” setting. We compared the anti-melanoma effect in the following three designs (Figure 7a): (1) Treatment on bottom of 12-well plate. The bottom of plate was the only barrier. Therefore, the total barrier thickness between CAP and cells was 1 mm. (2) Treatment on a polystyrene lid, which was just above the bottom of plate, with an air gap of 2 mm. Because of the existence of lid and plate’s bottom, the total thickness of barrier was 4 mm. (3) Here, 20 mL (thickness 2 mm) of PBS (Gibco, 10010023) was added in the polystyrene lid. 

Both polystyrene material and a small air gap (2 mm) did not block the physical effect of CAP on B16F10 cells. Although these physical barriers did weaken the killing effect on cancer cells, a strong growth inhibition could also be observed (Figure 7b). A layer of PBS could completely inhibit the physically based cell death. Bright-field imaging was performed using a Nikon TS100 inverted phase-contrast microscope. As shown in Figure 7c, typical physically triggered necrosis could not be observed when a short (0.5 min) treatment was performed using a ‘barrier+’ design. However, such a necrosis feature could be widely observed when treatment extended more than 1 min in both ‘barrier−’and ‘barrier+’ design. 

## 3. Discussion

As compared with chemically based CAP treatment, physically based CAP treatment has diverse unique features and advantages. First, physically based treatment does not rely on reactive species. Thus, it can effectively kill reactive species-resistant cells such as the B16F10 cells in this study [26]. Furthermore, physically based treatment has a unique transbarrier killing effect, which is due to the nature of EM effect. The discovery of such transbarrier capability provides a novel clue to understand the transdermal antitumor effect of CAP by just treating skin above the subcutaneous tumorous tissue [27,28]. Additionally, probably due to spatial and strength distribution of the EM effect from CAP, the biological effect of physically based treatment is highly sensitive to the relative position between plasma and targets. For instance, as we demonstrated in Figure 2b, the contact between one millimeter scale of the CAP jet’s tip with a physical barrier could kill nearly all cells in a circle with several centimeters scale simultaneously. All these features clearly suggest that there are plenty of unknowns about using CAP’s physical effect in medicine.

The effective delivery of CAP’s antitumor factors to affect corresponding tissues is a challenge in plasma medicine. Given such a significance, the contactless anticancer effect should capture more attention. In this study, we have demonstrated that the EM effect of CAP could strongly kill B16F10 cells after it passed a 6 mm thick glass and 1 mm thick polystyrene material. Even an air gap of 2 mm sandwiched by two 1 mm thick polystyrene materials would not stop the penetration of physical effect on B16F10 cells. These preliminary studies provide important guidelines to design novel CAP sources using their EM effects. Glass or other transparent dielectric materials have been widely used in building CAP sources, particularly novel sources such as discharge tubes [29]. The contactless anti-melanoma capability provides promising hope to use CAP as a non-invasive cancer therapy modality, which may be the first step to use CAP in deep tumorous tissue treatment.

A suitable range of operational parameters is necessary to observe physically based anticancer performance. Actually, it is also necessary to find a suitable condition to observe chemically based anticancer performance, such as using the CAP-activated solution or medium [30,31,32]. Here, flow rate has been found as a pivotal parameter to modulate physical effect. In fact, a flow rate in a middle range rather than the maximum value can result in the maximum killing effect. Moreover, the size of copper sheet between CAP and the target can also modulate the killing effect. Given this discovery, a reasonable design of a copper pad may guide the physical effect to merely affect tumorous tissues and protect normal tissue areas by covering them. 

Understanding the mechanism should be a main challenge in the future. So far, though numerous observations about physically based anticancer capacity have been published, the underlying mechanism is far from clear. What is the effective frequency of the EM effect from CAP? What is the spatial distribution of such an EM effect? Can we achieve a better penetration depth of EM effect using novel designs? Will such an effect also work in vivo? The answers to these questions will build a cornerstone of the wide application of physically based CAP technology in medicine.

## 4. Methods and Materials

### 4.1. Cell Culture

The cell culture medium was composed of RPMI-1640 (ATCC 30-2001) supplemented with 10% (*v*/*v*) FBS (GE Healthcare, Silver Spring, MD, USA) and 1% (*v*/*v*) penicillin/streptomycin solution (Life Technologies, Frederick, MD, USA). A murine melanoma cell line B16F10 was cultured under standard conditions (a humidified, 37 °C, 5% CO_2_ environment). 

### 4.2. CAP Jet Source

**Figure 8:**
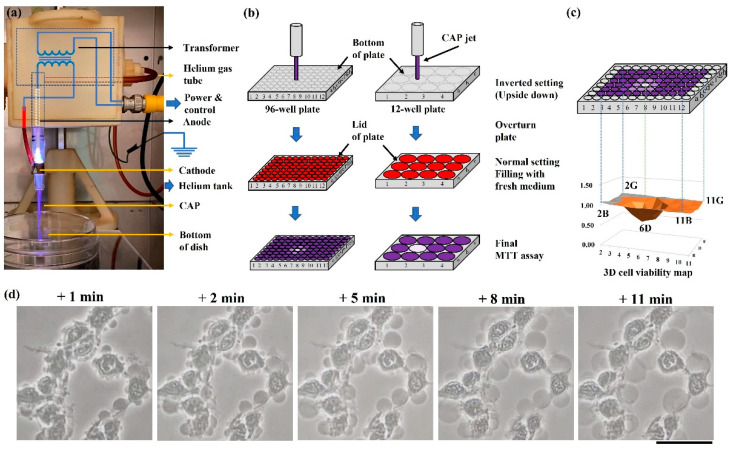
The CAP jet source and physically based treatment: (**a**) The CAP jet source; (**b**) basic strategies to perform a physically based treatment on 96-well or 12-well plates; (**c**) 3D cell viability map. The 3D map presents all middle 10 × 6 wells’ relative cell viabilities in one figure. Five marked well numbers represent the boundary wells and the CAP-treated well “6D”. (**d**) A time-lapse imaging of a typical cellular change over the initial 11 min after a 2 min of treatment. Photos were taken at 1, 2, 5, 8, and 11 min after treatment, using a Nikon TS100 inverted phase-contrast microscope. Scale bar = 50 μm.

In this study, the CAP source was a typical CAP jet source (Figure 8a). Discharge in helium (99.995% purity, Roberts Oxygen, grade 4.5) was triggered by an alternating voltage (~3 kV, peak value) between one central anode and another annulus cathode close to the nozzle. The ionized gas flowed away from the main discharge arc area and immediately entered air containing N_2_ and O_2_. Finally, a violet jet-shaped gas matter that contained plenty of reactive species and other chemical components flowed out the nozzle. Helium flow rate was modulated by a flow meter. The CAP-treated surface’s temperature was less than 40 °C after a treatment lasting 8 min.

### 4.3. Physically Based CAP Treatment

CAP simultaneously generates chemical and physical factors offering a multimodal opportunity. To study the physical effect in CAP, all chemical factors, particularly, reactive species should be blocked or filtered. To achieve this goal, we proposed the following strategies to perform physically based treatment by blocking chemical factors, which naturally realized a contactless treatment between CAP and cancer cells (Figure 8b): For the widely used polystyrene multi-well plates or dishes, bottom thickness or lid thickness was 1 mm. Physically based treatment can be performed on the bottom of an inverted (upside-down) dish or plate, which is the easiest way to observe a strong physically triggered effect. Additionally, CAP treatment can be performed on the dish or plate as long as the gap between lid (physical barrier) and bottom is not too large. Here, we performed the physically based treatment both ways. 

As an example, we illustrated the protocols to perform physically based treatment on the bottom of an inverted 96-well or 12-well plate. For the 96-well plate, the overnighted supernatant was removed before the treatment. Due to the surface tension, there was still a thin layer of medium to cover cells, particularly, at the junction between the wall and bottom of each well. Such an inverted setting for 10–20 min would not cause observable impact on the cell viability [16,17,18]. A single treatment was performed at the well “6D”. The EM effect in CAP would affect several surrounding wells. After treatment, 100 μL/well medium was immediately supplied to all middle 10 × 6 wells. Cells were further cultured one day before final viability assay. For the 12-well plate, a single treatment was only carried out on one well in the middle two columns. All wells in the peripheral columns were used as the control group. After treatment, 1 mL/well medium was immediately supplied to culture cells for three days before ultimate viability assay. 

Following standard protocols, cell viability assay was carried out using MTT assay (Sigma-Aldrich, M2128). Absorbance (570 nm) was measured using a microplate reader (Hybrid Technology). To obtain the relative cell viability, all data were processed by the division between the experimental group and the control group. The experiments were repeated at least three times. By using Origin (OriginLab Corporation, Northampton, MA, USA), statistical significance was calculated via one-way analysis of variance (ANOVA) followed by Bonferroni post hoc test for multiple comparisons.

For the 96-well plate, because the EM effect in CAP would affect several surrounding wells, even the treatment was only performed on a single well, the final cell viability was presented in a 3D cell viability map. As shown in Figure 8c, the obtained cell viability values in all middle 10 × 6 wells were divided by the control group’s value. The obtained relative cell viabilities in all 10 × 6 wells were presented in a single 3D cell viability map. 

### 4.4. Basic Necrotic Features

The B16F10 cells showed obvious necrotic features after treatment. A time-lapse imaging is presented in Figure 1d. Cytosol aggregation and strong bubbling on the cellular membrane were two main features. Cytosol aggregation finished just during the CAP treatment. The bubbling was due to the leakage of cellular solutions into the extracellular environment. Bubbles should have a membrane as the interface to distinguish them from the extracellular environment and probably just to be a part of the cytoplasm membrane. Hours after treatment, most bubbles detached or disappeared, leaving ‘empty shell-shaped’ dead cells, which were the representative features in the microscopic imaging days after treatment. 

## 5. Conclusions

A CAP jet is a promising anti-melanoma modality. So far, the use of CAP sources has mainly been limited to a chemically based approach. Here, we demonstrated the use of a CAP jet in a contactless way, which was fully determined by the EM effect generated in the CAP jet. The flow rate of noble gas could modulate physically based growth inhibition on melanoma cells. Physically based treatment could achieve a strong anti-melanoma efficacy; even plasma was blocked from cells by dielectric barriers, such as polystyrene and glasses with the largest total thickness of 7 mm. Furthermore, water could counteract the physical effect of the CAP jet. These discoveries build guidelines for designing novel CAP sources such as discharge tubes and the use of the EM effect from CAP sources in modern medicine, may have a profound impact on plasma medicine. 

## Data Availability

The data presented in this study that support the findings are available on reasonable request from the corresponding author.
